# The Military Position of the Medical Student in Great Britain and in America

**Published:** 1917-10-20

**Authors:** 


### THE MILITARY POSITION OF THE MEDICAL STUDENT IN GREAT BRITAIN AND IN AMERICA.

### A Contrast in Policy and Methods.

The war is already bringing to the United States experiences similar to those which in its earlier days it brought to Great Britain, and the process is sure to be repeated. It will be of interest to watch how these experiences will be met. The observation will certainly not be a cold, critical, and detached one. Rather it will be associated with the utmost degree of sympathy, goodwill, and a desire to help. Such, of course, comes within the claim of comradeship, and especially of comradeship in a common purpose. Moreover, there is not wanting a strictly practical motive. What will help and hasten the preparations of our Transatlantic Allies will go to contribute to their full and prompt striking power—in other words, to the shortening of the war and to the saving of our own skins. We may, therefore, on the grounds both of duty and of interest gladly place at the disposal of our comrades and friends whatever we have learned in the stern and sad experiences of the past three years. A cheap and superficial proverb announces that experience teaches fools. But this, we venture to suggest, is a misreading of the lesson of life. Experience teaches and makes wise men. The fools will not learn from any discipline. If we are sadder men than we were three years ago we are also wiser, and if our added wisdom will help those who are making ready to help us and the cause for which they and ourselves both stand, it is a natural and becoming thing to add it to the common stock. The giving will have no touch of arrogance, but will be an index of the mutual service and cooperation which we earnestly desire to cultivate, and which will help to strengthen ties that have world welfare for their motive and sanction.

### America's National Characteristics.

Nor need there be any fear that the receiving will show a less worthy spirit than the giving. Between generous minds embarked on a common enterprise there is no question of a market. Who gives and who takes matters not so long only as the end is served, and a ledger account is for the petty and the calculating. Those who cannot welcome the word and hand and purse of a friend may be for the lonely furrow, but have no place in the roll of comradeship. America—all the world knows it—is not of this spirit. She will give with a free hand and a generous heart, and equally she will accept whatever help may come to her from those who are united with her in loyal purpose and resolve. No petty vanity or puerile sensitiveness will mark the course of her dealings, and, what is more, no considerations of this order will even touch her mind. By a high-minded and generous nation such influences would be judged as unworthy, and an eminently practical and common-sense people would regard them as ridiculous. It is not only in material but in mind that America has united herself to the nations set on a change in the world order, and what she or others may contribute is equally at the service of all. Let no one imagine that Americans have any measure of that thin and empty pride which prefers to be ignorant rather than to be instructed by others. On the contrary, their eager and quick receptivity and their alert attitude towards new ideas are among their most impressive national characteristics, and these will not fail them on the present occasion. All this might have been said as a matter of anticipation. To-day it is a chronicle of actual experience, more especially for those who have been allowed to get a little distance behind the scenes. The gathered lessons of the past three years are being carefully studied by quick and reflective minds, and they are welcomed from all sources. The givers will get more than gratitude. What is received will be touched with a new genius, and the teachers may quite well find their instructions bettered. Just now we who have been chastened in the school of experience have some precepts to impart, and we may be sure of a willing audience. When our turn comes to listen—as doubtless it will come—we shall hope to listen with an equally good grace. In the meantime some blunders and miscalculations have given us warning and wisdom, and the fruit of these is freely at the service of our friends.

### Problems to Face and Errors to Avoid.

Necessarily in these columns we follow with a special interest the arrangements which are being made for the organisation of the medical work of the war. The problems which present themselves in this direction include not only the supply of doctors to the Army, but also the maintenance of an adequate medical service for the civil population, and the reconciliation of these two claims has caused many difficulties. Indeed, we can hardly pretend that even yet this reconciliation has been satisfactorily effected. Doubtless here, as elsewhere, the situation has been complicated by an error of judgment relative to the length of the war. It is true that at the outset an authoritative voice warned us that our calculations ought to be made on the basis of a three years' struggle. But this was generally regarded as an outside estimate, and was by no means accepted as a probable happening. Yet we are now in the fourth year of fighting, and the end is not yet. Then, in the earlier days, the voluntary principle held undisputed sway, and every individual was free to frame his own judgment and decide his own action. It took both time and experience to convince our people that the situation could not be fully met in this fashion. The sacrifice of a long-standing national tradition was not an easy matter, and we have had to pay for our education. Our friends on the other side of the Atlantic

THE MEDICAL STUDENT IN GREAT BRITAIN AND AMERICA—(*continued*).

are not going to be hindered by either of these considerations. They are making sure preparations on the view that the task is going to be both difficult and prolonged, and knowing the troubles which our own errors have cost us, we can but rejoice at this decision. Again, their President took from the outset a strong stand on the question of compulsion, and held that in a great national crisis it is the community and not the individual himself who must determine the citizen's duty. These two influences will play an important part in the American preparations, and the nation will thus avoid many mischiefs which we ourselves have suffered. There is a clear line of prescribed duty for each person concerned, and thus individuals are spared doubts and hesitations and uncertainties, while the authorities have a confident knowledge of the resources on which they can count. Our own people have had to go through an exactly contrary order of experience, and the effect of this may even yet be detected in our arrangements.

### Too Many Directions.

We may see an illustration of the two atmospheres thus contrasted in the position occupied by the medical students of the two countries. Here, in the early days, the students in large numbers, as was natural, joined the combatant ranks of the Army. Then it became evident that this proceeding would have a fatal effect on the immediate supply of new practitioners, for among the volunteers were many who had reached the fourth or fifth year of the curriculum. These, therefore, had to be brought back to their studies, and many of them are by this time experienced and competent military surgeons. But while the seniors were enjoined to continue their studies and to secure a legal qualification at the earliest possible moment, the juniors were left without direction; or, more strictly speaking, they were left with too many directions. On one side were voices urging them to enter the combatant ranks, and on the other equally authoritative counsels advised prosecution of their medical studies. The unhappy student had a thoroughly bad time of it, and our medical schools were the scenes of much agitated debate. When the date of compulsory service arrived, the students, like their fellows in the non-medical world, came under its provisions, and the numbers in attendance at the schools suffered heavily. Now the senior men are allowed to go on to the end of the curriculum and to the formal diploma, in the expectation that they will take commissions in the R.A.M.C. But the juniors come into the grasp of the recruiting officer. If they are marked in Class A there is no question, and more recently those put in Class B have been called up, while lads formerly rejected have been re-examined, and many of them have been grouped as fit for one or other of the formal categories. The medical student world during the last three years has been anything but a happy and confident one, and even now stability does not mark it for its own.

### The Future Effect of Military Demands.

Still more important is the effect which the military demands must inevitably exercise on the supply of medical practitioners in the immediate future. The process has been going on for three years, and the consequences are now at our doors. Suppress the medical student to-day and there must needs be a gap in the ranks of the practitioners in five years' time. Further, there is no possible emergency contrivance which will meet the defect, seeing that the educational preparation cannot possibly be curtailed, and no one would propose that groups of half-trained doctors should be let loose upon the community. These are the cold, hard facts of the situation, and no smooth words will conjure them away. The present year may perhaps see additions to the profession at about the average level, but in the three following years there will be a considerable deficiency, even when due allowance is made, numerically and otherwise, for a larger number of women practitioners. At the same time the needs of the Army, the casualties of war, and the extended medical necessities of the community call for additions not merely up to but above the average. In short, we have to meet a situation in which an enlarged demand is associated with a falling supply. To look back on the past for opportunities for censure and blame is an idle task, and we are not going to pursue it. But the present is with us, and we may attempt to redeem the future. The war is not ended, nor will the demands which directly arise from it promptly cease with the proclamation of peace. Our civilian population is already suffering from a reduction in the provision of medical services, and here it must be said that the limit has been reached. The needs of the immediate future in this respect are urgent.

### Prompt Action Called For.

In view of all these circumstances it is imperative that medical students serving in the Army shall be ordered to return to their studies, and shall be instructed that their proper contribution to the national cause is to pursue these studies with zeal, so as to obtain at the earliest possible moment a full legal qualification. If any on due trial should prove themselves lazy or incompetent they can be recalled to active service, and provision can easily be made to prevent a pretence of medical study being utilised as a shelter for the faint-hearted. The war has proved the necessity for the cultivation of foresight and the long view. This necessity still exists. In the particular direction here contemplated it has a most emphatic importance, for unless we cultivate the medical student to-day we shall look in vain for the medical practitioner of to-morrow, and the failure must have serious consequences for the protection and welfare of the community. There is perhaps just time to keep this failure within manageable proportions. But in order to do so action must be prompt and firm and inclusive.

### THE MEDICAL STUDENT IN GREAT BRITAIN AND AMERICA— (*continued*).

### What the U.S.A. Has Done.

A very different picture comes from the other side of the Atlantic. Whether taught by the predicament in which we at present find ourselves, or warned by native shrewdness, the people of the United States are not going to permit any risk of a short supply of medical practitioners. For a time the promise was quite the other way. Medical students and hospital residents came like their fellows under the incidence of the draft, and had this been continued the selective law would have taken its quota from their numbers. What has happened in this country would have been repeated in America. There is no need to blame the military authorities of either country. Their duty is to find the men needed for the Army, and they can scarcely be expected to recognise without aid all the refinements of the situation. Fortunately such aid was promptly forthcoming, and it did not lack vigorous or tactful exponents. The New York *World* commenced an energetic agitation for the exemption of medical students, and it was supported by a committee of the hospital and medical faculties under the chairmanship of Dr. S. S. Goldwater. In characteristic fashion circular-letters and telegrams were sent in every direction, and within a few days —they cultivate dispatch in the States—nearly every medical college in the country, together with the hospitals and the medical journals, were bombarding the central authorities with figures and estimates showing that unless medical students were exempted from the draft the mistakes and mischiefs suffered by England and France would be repeated, and the country would inevitably suffer from a short supply of doctors. We are even informed that the British Army medical authorities rallied to the movement, though we publish the announcement with all due reserve. Accepting it as true, we may surely look for works of repentance nearer home.

### Ample Supply of Practitioners Secured.

The strength of public opinion aroused by the agitation has its measure in a very striking incident. Certain local boards in Cleveland and St. Louis practically took the law into their own hands, and granted exemptions to medical students when strictly speaking such a course was quite illegal, and their example seemed likely to spread. What penalties were risked by this proceeding we cannot pretend to know, but happily all debate on this issue is unnecessary, for wisdom under increasing pressure awoke at the centre, and Washington decreed the exemption demanded. All students who have completed their first year are freed from the incidence of the selective draft, and are directed to continue their studies and to take their diplomas. Similar conditions will apply to hospital residents or internes, with such limitations and qualifications as the interests of the country demand, and both the one group and the other will be enrolled in the Reserve Corps of the Medical Department. The details of the arrangements need not be set forth here, but the broad effect of them is to maintain the numbers of medical students on the ground that an ample supply of practitioners in the immediate future is an essential interest of the nation. Needless to say we heartily concur, and we would fain hope that the force of the example may reach our own shores. The result has not been gained without effort, and we will venture to offer our hearty congratulations to our contemporary the New York *World* on the insight, energy, and enterprise which have marked its conduct of an agitation directed to a lofty end and dictated by motives of the highest patriotism.

